# Multi-Target Protective Effects of β-Caryophyllene (BCP) at the Intersection of Neuroinflammation and Neurodegeneration

**DOI:** 10.3390/ijms26136027

**Published:** 2025-06-23

**Authors:** Caterina Ricardi, Anna Mazzierli, Stefano Guglielmo, Nicola Origlia, Francesca Gado, Clementina Manera, Grazia Chiellini, Beatrice Polini

**Affiliations:** 1 Department of Pathology, University of Pisa, 56126 Pisa, Italy; c.ricardi@student.unisi.it (C.R.); a.mazzierli1@student.unisi.it (A.M.); 2 BIO@SNS Laboratory, Scuola Normale Superiore, Via Moruzzi 1, 56124 Pisa, Italy; stefano.guglielmo@sns.it; 3 Institute of Neuroscience, Italian National Research Council (CNR), 56124 Pisa, Italy; origlia@in.cnr.it; 4 Department of Drug Sciences, University of Pavia, 27100 Pavia, Italy; francesca.gado@unipv.it; 5 Department of Pharmacy, University of Pisa, 56126 Pisa, Italy; clementina.manera@unipi.it

**Keywords:** beta-caryophyllene, CB2R, PPARγ, neuroinflammation, microglia, BDNF, LTP

## Abstract

Recent advances in cannabinoid-based therapies identified the natural CB2 receptor agonist β-caryophyllene (BCP) as a promising anti-inflammatory and neuroprotective agent. To further explore its therapeutic potential on the management of neurodegenerative disorders, in the present study we investigated the ability of BCP to prevent neuroinflammation and promote neuroprotection by using both in vitro and ex vivo models of β-amyloid induced neurotoxicity. Our data showed that BCP significantly protected human microglial HMC3 cells from Aβ_25-35_-induced cytotoxicity, reducing the release of pro-inflammatory cytokines (TNF-α, IL-6) while enhancing IL-10 secretion. These effects were associated with a reduced activation of the NF-κB pathway, which emerged as a central mediator of BCP action. Notably, the use of CB2R- or PPARγ-selective antagonists revealed that the observed NF-κB inhibition by BCP may involve the coordinated activation of both canonical (e.g., CB2R) and non-canonical (e.g., PPARγ) receptors. Moreover, BCP restored the expression of *SIRT1*, *PGC-1α*, and *BDNF*, indicating the involvement of neurotrophic pathways. Clear neuroprotective properties for BCP have been highlighted in Aβ_1-42_-treated brain slice preparations, where BCP demonstrated the rescue of both the amyloid-dependent depression of BDNF expression and long-term synaptic potentiation (LTP) impairment. Overall, our results suggest that BCP constitutes an attractive natural molecule for the treatment of Aβ-induced neuroinflammation and synaptic dysfunction, warranting further exploration for its clinical application.

## 1. Introduction

The endocannabinoid system (ECS) is a complex cell-signaling network involved in maintaining physiological homeostasis, particularly through the regulation of immune responses, inflammation, and neuronal function. It comprises endogenous lipid-based ligands (endocannabinoids), enzymes responsible for their synthesis and degradation, and two primary G protein-coupled receptors: cannabinoid receptor 1 (CB1R) and cannabinoid receptor 2 (CB2R). Initially, CB1R was primarily associated with the central nervous system (CNS), whereas CB2R was considered to be limited to peripheral immune tissues. However, subsequent evidence has revealed the widespread expression of both receptors in central and peripheral tissues. In particular, CB2Rs are predominantly expressed in cells of the immune system—such as macrophages, leukocytes, spleen, and tonsils—where they modulate cytokine release and immune cell trafficking [1,2]. Recent studies have confirmed CB2R expression within the CNS, underscoring its emerging relevance in neurological processes and diseases [3].

Due to their immunomodulatory and neuroprotective properties, selective CB2R agonists have attracted growing interest as potential therapeutic agents, particularly in the context of neurodegenerative diseases (NDDs) [4]. NDDs are multifactorial conditions characterized by the progressive degeneration of specific neuronal populations, leading to impairments in cognitive, sensory, and motor functions. Alzheimer’s disease (AD) is a leading cause of dementia, pathologically characterized by progressive synaptic degeneration, brain atrophy, extracellular amyloid-beta (Aβ) plaque deposition, and neurofibrillary tangles composed of hyperphosphorylated tau. Neuroinflammation, primarily mediated by activated microglia, plays a central role in disease progression and has been strongly implicated in synaptic dysfunction [5,6,7]. Current pharmacological approaches targeting single pathological mechanisms have had limited clinical success, highlighting the value of multi-targeted therapies. Consequently, modulation of the ECS, and, in particular, of CB2R, offers a promising strategy, as shown by preclinical models reporting encouraging results with CB2R-selective ligands [8].

Among the CB2R agonists under investigation, beta-caryophyllene (BCP), a natural bicyclic sesquiterpene present in many essential oils, has emerged as a particularly promising candidate. BCP selectively activates CB2R without eliciting CB1R-mediated psychoactive effects, and its pharmacological profile is characterized by anti-inflammatory and antioxidant activities. In vitro and in vivo studies have demonstrated that BCP can attenuate oxidative stress, preserve mitochondrial integrity, and reduce amyloid burden [9,10]. CB2R activation by BCP has also been associated with improved cognitive function and attenuated neuroinflammation in animal models of AD [9,11]. Furthermore, BCP was the first natural CB2R agonist to show efficacy in models of inflammatory and neuropathic pain [12], and its therapeutic potential extends to metabolic disorders, including obesity, NAFLD/NASH, diabetes, and cardiovascular diseases [13]. These systemic effects are largely attributed to its ability to inhibit key pro-inflammatory mediators such as NF-κB, cytokines, chemokines, and adhesion molecules, as well as its modulation of signaling pathways including opioid receptors, sirtuin 1/PGC-1α, and activation of nuclear PPARs—key regulators of glucose and lipid homeostasis and inflammation [14,15]. Several studies reported a great link between CB2R activation and PPAR-γ receptor stimulation [16,17,18,19]. In this regard, BCP has been shown to exert anxiolytic, antioxidant, and anti-inflammatory effects thanks to PPAR-γ activation following CB2R stimulation [14,20].

Notably, BCP is recognized as safe by the FDA and approved as a food additive and preservative, with a favorable toxicological profile and no significant adverse effects reported [21]. On these premises, our study aims to investigate the ability of BCP to modulate neuroinflammatory responses and exerting neuroprotective effects. Specifically, we assessed its activity by using both in vitro and ex vivo models of neuroinflammation and Aβ-induced neurotoxicity. Our results indicated that BCP exerts anti-inflammatory and neuroprotective effects through a CB2R–PPARγ-mediated pathway, which leads to NF-κB inhibition contributing to anti-inflammatory responses and to the preservation of brain-derived neurotrophic factors (BDNFs) production, which plays an important role in maintaining synaptic plasticity in learning and memory, with significant implications for NDD treatment.

## 2. Results

### 2.1. BCP-CB2R System Protects HMC3 Cells from Aβ_25-35_-Induced Cytotoxic Effect

Microglia cells serve as the primary immune defense within the CNS, and their dysfunction is recognized as a key contributor to the pathophysiology of various NDDs [22,23].

**Figure 1:**
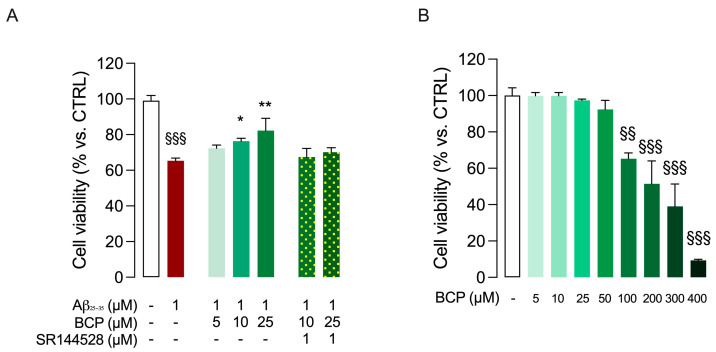
BCP prevents Aβ_25-35_-induced cytotoxicity in HMC3 cells. As shown in (**A**), HMC3 cells were initially treated with increasing concentrations of BCP (5, 10, 25 µM) for 24 h and subsequently exposed to 1 µM Aβ_25-35_ for 48 h. Cell viability was quantified by MTT assay. To assess whether the cytoprotective effect observed when BCP was used at 10 and 25 µM concentrations could involve a CB2R-mediated response, co-administration experiments with the CB2R-selective antagonist SR144528 (1 µM) have also been performed. In addition, HMC3 cells were treated with BCP (5–400 µM) for 24 h to test its cytotoxicity by viability assays (**B**). In all the experiments data represent means ± S.E.M. from three independent experiments (*n* = 3), performed in triplicate. Statistical analysis was performed using ordinary one-way ANOVA followed by Tukey’s (**A**) or Dunnet’s (**B**) multiple comparison test. ^§§^ *p* < 0.01, ^§§§^ *p* < 0.005 compared to control cells; * *p* < 0.05, ** *p* < 0.01 compared to cells exposed to Aβ 1 μM treatment for 48 h.

To investigate the potential protective activity of BCP against Aβ_25-35_-induced cytotoxicity, HMC3 cells were exposed to pretreatment (24 h) with increasing concentrations (5, 10, 25 µM) of BCP before being treated for 48 h with 1 µM Aβ_25-35_ peptide (Figure 1A). Of note, no cytotoxic effects were detected in HMC3 cells after treatment with BCP at 5, 10, and 25 μM, whereas when used above 50 μM, a low inhibition of cell viability started to be observed (Figure 1B).

Exposure to Aβ_25-35_ (1 μM, 48 h) significantly decreased HMC3 cells viability, and this cytotoxic effect was significantly attenuated by pretreatment with BCP (5, 10, 25 μM; 24 h) in a concentration-dependent manner. In addition, pretreatment with the CB2R selective antagonist SR144528 (1 μM) completely abolished the protective effect of BCP, suggesting a CB2R-mediated action (Figure 1A).

### 2.2. BCP-CB2R System Protects HMC3 Cells from Aβ_25-35_-Induced Pro-Inflammatory Response

Aβ peptides can activate microglia, triggering the release of numerous pro-inflammatory mediators [24,25]. Consequently, suppressing the microglial response to inflammatory stimuli could represent a potential strategy to delay the onset of AD and potentially slow its progression [22,26,27]. Therefore, HMC3 cells were used to explore the effects of BCP against Aβ- induced inflammation. Since BCP at 10 and 25 µM effectively protected HMC3 cells from Aβ-induced cytotoxicity, these concentrations were used to assess its ability to counteract Aβ-induced inflammatory response.

**Figure 2:**
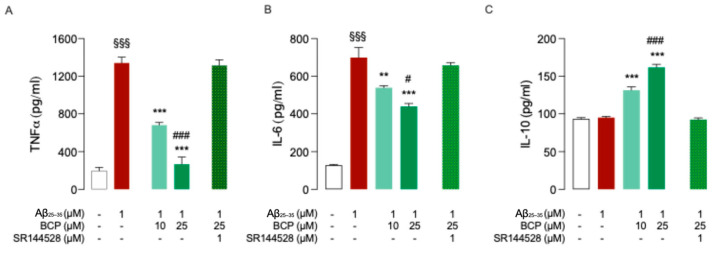
BCP prevents Aβ_25-35_-induced inflammatory response in HMC3 cells. BCP pretreatment prevents the inflammatory response of HMC3 cells exposed to Aβ_25-35_ 1 μM for 48 h, and bars indicate the release (pg/mL) of tumor necrosis factor alpha TNFα (**A**), IL-6 (**B**), and IL-10 (**C**). In all experiments, pretreatment with SR144528 (1 μM) completely abolished the effect produced by BCP used at 25 μM concentration (**A**–**C**). In all the experiments data represent means ± S.E.M. from three independent experiments (*n* = 3), performed in duplicate. Statistical analysis was performed by ordinary one-way ANOVA followed by Dunnet’s multiple comparison test. ^§§§^ *p* < 0.005 compared to control cells; ** *p* < 0.01 and *** *p* < 0.005 compared to cells exposed to Aβ 1 μM treatment for 48 h; ^#^ *p* < 0.05 and ^###^ *p* < 0.005 compared to cells treated with BCP (10 µM, 24 h) before exposure to Aβ_25-35_ (1 μM, 48 h).

As revealed by ELISA assays, exposure of HMC3 cells to 1 μM Aβ_25-35_ for 48 h promoted a significant increase in the secretion of common pro-inflammatory cytokines TNF-α and IL-6 (Figure 2A,B) without affecting the release of anti-inflammatory cytokine IL-10 (Figure 2C). Pretreatment with 10 and 25 μM BCP showed a significant dose-dependent reduction in TNF-α and IL-6 levels (Figure 2A,B), along with a dose-dependent increase in IL-10 secretion (Figure 2C).

Co-administration of the CB2R-selective antagonist SR144528 (1 μM) abolished the BCP protective effect against Aβ-induced microglia activation (Figure 2A–C), confirming the involvement of CB2R in mediating these responses.

To better understand the mechanisms underlying the anti-inflammatory effect exerted by BCP in Aβ-induced HMC3 cells, we next investigated its ability to modulate the NF-κB pathway, a key regulator of glial activation and cytokine production. The dysregulation of the transcription factor NF-κB has been widely associated with AD, as it promotes glial activation and sustained neuroinflammation [27]. Notably, inactivation of microglial NF-κB has been shown to restore cognitive function and shift microglia toward a homeostatic phenotype [28]. Therefore, we evaluated whether BCP could modulate NF-κB signaling in Aβ-treated HMC3 cells.

**Figure 3:**
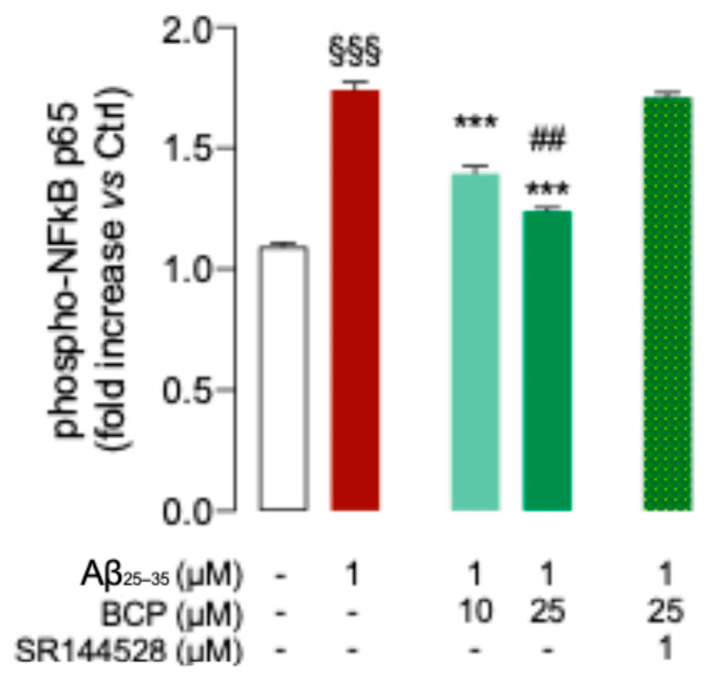
The BCP/CB2R system modulates the phosphorylation of P65 in Aβ_25-35_-induced HMC3 cells. BCP pretreatment suppresses the phosphorylation level of P65 in HMC3 cells exposed to Aβ_25-35_ 1 μM for 48 h. Pretreatment with SR144528 (1 μM) completely abolished BCP effect. Data represent means ± S.E.M. from three independent experiments (*n* = 3), performed in duplicate. Statistical analysis was performed using ordinary one-way ANOVA followed by Tukey’s multiple comparison test. ^§§§^ *p* < 0.005 compared to control cells; *** *p* < 0.005 compared to cells exposed to Aβ 1 μM treatment for 48 h; ^##^ *p* < 0.01 compared to cells pretreated with 10 µM BCP before exposure to Aβ 1 μM.

Results showed that Aβ_25-35_ treatment enhanced phosphorylation level of P65 subunit, indicating activation of the NF-kB pathway, while BCP suppressed this response in a dose dependent manner (Figure 3). Notably, co-treatment with the CB2R selective antagonist SR144528 (1 μM) completely abolished the inhibitory effect of BCP on NF-κB activation (Figure 3), further confirming CB2R involvement.

**Figure 4:**
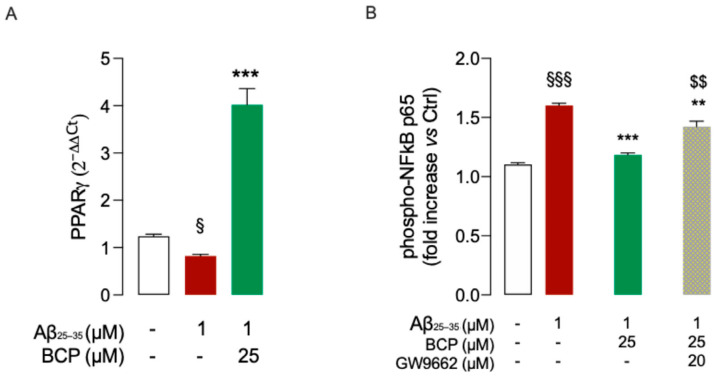
BCP counteracts Aβ-induced downregulation of *PPARγ*. Pretreatment with 25 μM BCP significantly counteracted the Aβ-induced the down-expression of *PPARγ* in HMC3 cells (**A**). In the presence of the selective PPARγ antagonist GW9662 (20 μM), the effect of BCP on reducing p65 phosphorylation in Aβ_25_-_35_-treated HMC3 cells (1 μM, 48 h) was partially abolished (**B**). Data represent means ± S.E.M. from three independent experiments (*n* = 3), performed in duplicate. Statistical analysis was performed using ordinary one-way ANOVA followed by Tukey’s multiple comparison test. ^§^ *p* < 0.05, ^§§§^ *p* < 0.005 compared to control cells; ** *p* < 0.01 and *** *p* < 0.005 compared to Aβ-treated cells; ^$$^ *p* < 0.01 compared to 25 µM BCP pretreated cells.

Based on previous evidence suggesting a functional interplay between CB2R and the nuclear receptor PPARγ [14,29], we next examined whether PPARγ might also contribute to the observed anti-inflammatory effect of BCP. After observing that Aβ exposure significantly reduced *PPARγ* expression in HMC3 cells (Figure 4A), we evaluated the potential effect of BCP pretreatment. Our results showed that BCP was able to prevent the Aβ-induced downregulation of *PPARγ* expression (Figure 4A).

To further explore the possible contribution of PPARγ activation to BCP’s anti-inflammatory action, we examined the effect of co-treatment with the PPARγ antagonist GW9662 (20 μM). Notably, GW9662 partially abolished the ability of BCP (25 μM) to prevent the Aβ-induced increase in p65 phosphorylation (Figure 4B), supporting the involvement of PPARγ in the anti-inflammatory mechanism of BCP.

Collectively, our data suggest the contribution of both CB2R and PPARγ activation on BCP anti-inflammatory action in HMC3 cells.

### 2.3. BCP Modulates BDNF’s Activity upon Activation of the PGC-1α and SIRT1 Pathways

BDNF is a key neurotrophin involved in neuronal survival, repair, synaptic plasticity, and cognitive function. Its expression is positively regulated by SIRT1 and PGC-1α, two key factors in mitochondrial function, oxidative stress response, and neuroprotection [30], which are also involved in the regulation of PPARγ expression and activity [31,32]. In detail, SIRT1 directly activates PGC-1α through deacetylation, enhancing its transcriptional activity [30], and this cooperative signaling contributes to BDNF upregulation [33].

**Figure 5:**
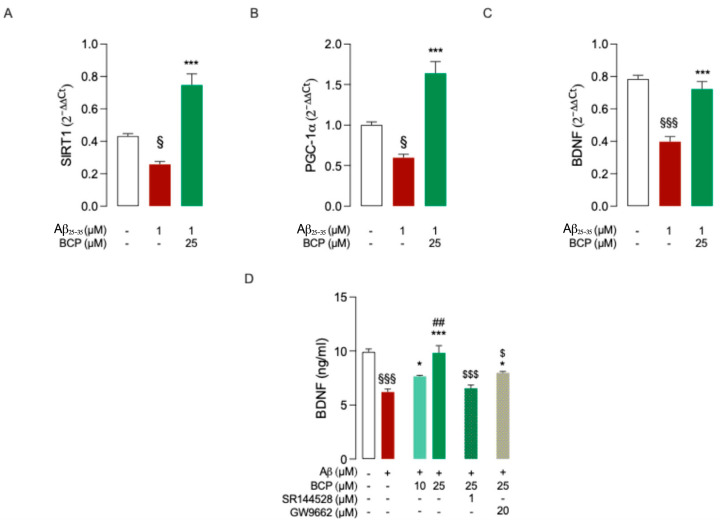
BCP enhances the expression of BDNF-related upstream regulators and restores BDNF levels in Aβ-treated HMC3 cells through the modulation of both CB2R and PPARγ signaling. Pretreatment with 25 μM BCP significantly increased the expression of *PGC-1α* (**A**), *SIRT1* (**B**), and *BDNF* (**C**) genes in HMC3 cells exposed to Aβ_25-35_ (1 μM for 48 h). In addition, BCP pretreatment increased BDNF protein levels in the same experimental conditions (**D**). Co-treatment with the CB2R antagonist SR144528 (1 μM) or the PPARγ antagonist GW9662 (20 μM) attenuated the effect of BCP (**D**). Data represent means ± SEM from three independent experiments (*n* = 3), performed in duplicate. Statistical analysis was performed using ordinary one-way ANOVA followed by Tukey’s multiple comparison test. ^§^ *p* < 0.05, ^§§§^ *p* < 0.005 compared to control; * *p* < 0.05, *** *p* < 0.005 vs. Aβ-treated cells; ^##^ *p* < 0.01 compared to 10 µM BCP pretreated cells; ^$^ *p*< 0.05 and ^$$$^ *p* < 0.005 compared to 25 µM BCP treated cells.

Therefore, to evaluate whether BCP could modulate this regulatory axis in Aβ-treated HMC3 cells, we analyzed the transcriptional expression of *SIRT1*, *PGC-1α*, and *BDNF*. Gene expression analysis revealed that exposure to Aβ_25-35_ led to a significant reduction in the transcriptional levels of *SIRT1*, *PGC-1α*, and *BDNF* in HMC3 cells. Pretreatment with 25 μM BCP effectively counteracted this downregulation and markedly increased the expression of all three genes (Figure 5A–C), suggesting that BCP may act through a *SIRT1/PGC-1α*-dependent mechanism to enhance *BDNF* expression. SIRT1 silencing experiments might help to clarify this aspect.

To confirm these findings at the protein level, BDNF concentrations were measured by specific ELISA assay. As expected, treatment with Aβ_25-35_ significantly reduced BDNF levels, whereas pretreatment with BCP (25 μM) restored them to near-control values (Figure 5D). Notably, the effect of BCP was reduced by co-administration of either CB2R-selective antagonist SR144528 (1 μM) or PPARγ antagonist GW9662 (20 μM), confirming the involvement of both CB2R and PPARγ in mediating this neurotrophic response in HMC3 cells (Figure 5D).

### 2.4. BCP Prevents Aβ_1-42_-Mediated Release of Pro-Inflammatory Factors in Mouse Organotypic Brain Slice Cultures (BSCs)

Given the promising results obtained by administering BCP to HMC3 cell cultures, we further explored the ability of BCP to target Aβ pathology using organotypic brain slice cultures (BSCs). BSCs constitute a physiologically relevant three-dimensional model of the brain [34]. A main advantage of BSCs is that they allow neuronal and non-neuronal cells from the brain to be studied in an anatomically preserved environment [34].

**Figure 6:**
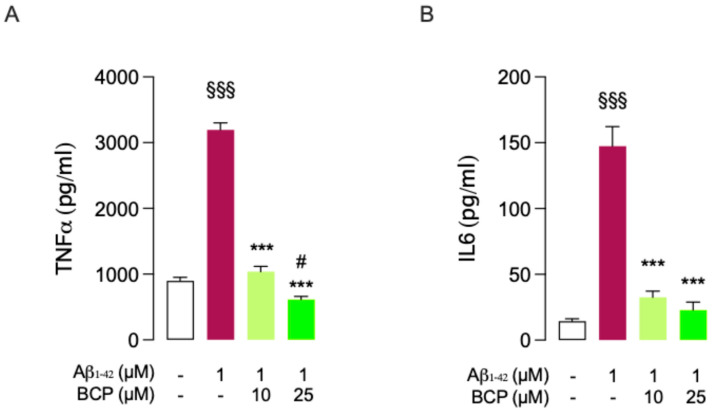
BCP prevents a Aβ_1-42_-mediated inflammatory response in mouse BSCs. BCP pretreatment prevents the inflammatory response caused by exposure to Aβ_1-42_ 1 μM for 24 h. Bars indicate the release (pg/mL) of TNFα (**A**) and IL-6 (**B**). In all the experiments data represent means ± S.E.M. from four independent experiments (*n* = 4), performed in duplicate. Statistical analysis was performed using ordinary one-way ANOVA followed by Tukey’s multiple comparison test. ^§§§^ *p* < 0.005 compared to control slices; *** *p* < 0.005 compared to slices exposed to Aβ 1 μM treatment for 24 h; ^#^ *p* < 0.05 compared to 10 µM BCP pretreated cells.

As revealed by ELISA assays, exposure of slices to 1 μM Aβ_1-42_ for 24 h promoted a significant increase in the secretion of common TNF-α and IL-6. Pretreatment with 10 and 25 μM BCP for 4 h almost completely abolished Aβ_1-42_ effect (Figure 6).

### 2.5. Protective Effect of BCP Against Aβ_1-42_-Induced Neurotoxicity: Modulation of BDNF Activity in Mouse BSCs

**Figure 7:**
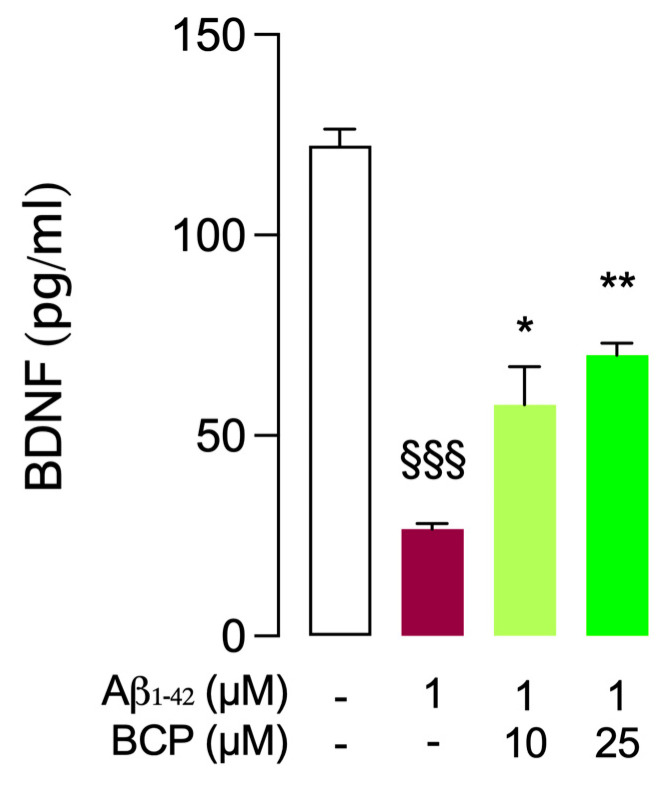
BCP restores Aβ_1-42_-dependent decrease in BDNF levels in mouse BSCs. As shown, 10 and 25 μM BCP pretreatment increases BDNF levels in mouse BSCs exposed to Aβ_1-42_ 1 μM for 24 h, and bars indicate the release (pg/mL) of BDNF. Statistical analysis was performed using ordinary one-way ANOVA followed by Tukey’s multiple comparison test. ^§§§^ *p* < 0.005 compared to control slices; * *p* < 0.05, ** *p* < 0.01 compared to slices exposed to Aβ 1 μM treatment for 24 h.

Aβ_1-42_ and BCP pretreatment experiments have been repeated to investigate the ability of BCP to modulate BDNF levels in mouse BSCs challenged with Aβ_1-42_. While BDNF levels were significantly diminished by treatment with Aβ_1-42_ (1 μM, 24 h), BCP (10 and 25 μM, 4 h) was able to significantly restore them in a dose-dependent manner (Figure 7).

### 2.6. BCP Acute Perfusion Counteracts Aβ_1-42_-Induced Inhibition of Long-Term Potentiation (LTP) in Mouse Brain Slices

**Figure 8:**
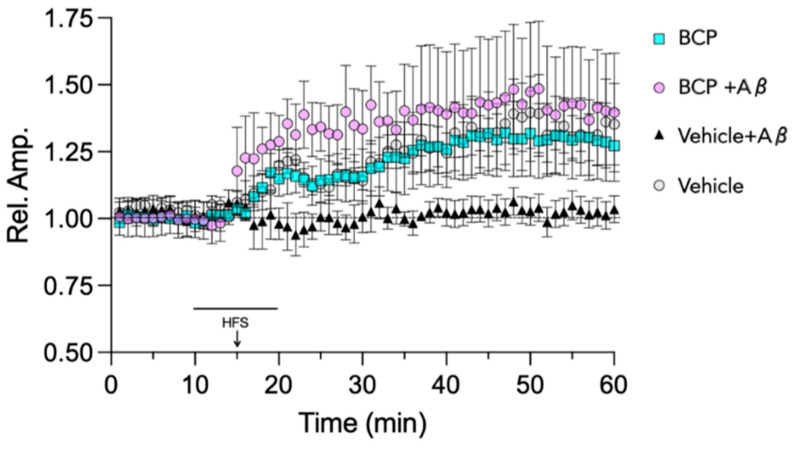
BCP rescues the inhibitory effect of Aβ_1-42_ on LTP in EC slices. BCP 10 µM was able to restore the synaptic plasticity impairment caused by acute perfusion Aβ_1-42_ 200 nM. High frequency stimulation (HFS, three trains of 100 pulses at 100 Hz, 10 s interval) was used to induce LTP. The LTP magnitude is expressed as relative amplitude (Rel. Amp.). Data points represent mean ± SEM of 6–10 slices per group, derived for at least 8 mice.

Building on encouraging results from previous experiments in BSCs, we next explored the ability of BCP to halt Aβ-induced synaptic impairment, focusing on the entorhinal cortex (EC), a brain area crucially involved in cognitive functions and highly susceptible to β-amyloid [35,36]. Specifically, the study examined the ability of BCP to counteract Aβ_1-42_-induced impairment of long-term potentiation (LTP), a fundamental neurophysiological mechanism for memory acquisition [37]. As shown in Figure 8, exposure to Aβ_1-42_ (200 nM, 10 min) significantly inhibited LTP at the level of the layer II of the EC, in agreement with previous findings [36,38]. Co-administration of BCP (10 μM) was able to rescue LTP inhibition induced by Aβ_1-42_ oligomers in the layer II of the EC. Notably, BCP used at 10 μM did not influence LTP when administered alone in control slices.

## 3. Discussion

In this study, we examined the neuroprotective proprieties of BCP against the deleterious effects of Aβ using both in vitro and ex vivo models of β-amyloid-induced neurotoxicity. Given the crucial role of microglia for the maintenance of CNS homeostasis, immune response and neural functions, we used human microglial HMC3 cells to assess the neuroprotective role of BCP. Since it is well known that CB2R activation switches microglia to an anti-inflammatory, pro-healing state and promotes migration to the site of the injury [39], we performed experiments on HMC3 cells to test the anti-inflammatory and neuroprotective activity of selective CB2R agonist BCP.

Our findings expand previously collected evidence [39,40,41,42,43], demonstrating that BCP significantly protected and rescued human microglia cells from the toxic effects exerted by exposure to β-amyloid. Specifically, we showed that pretreatment with BCP was able to suppress Aβ-induced release of TNFα and IL-6, while increasing the secretion of anti-inflammatory IL-10, indicating an anti-inflammatory response. Notably, these effects were completely abolished by co-treatment with CB2R antagonist SR144528, confirming the involvement of CB2R activation in BCP anti-inflammatory response.

CB2R activation is known to reduce neuroinflammation primarily by inhibiting adenylate cyclase and suppressing downstream pathways, including NF-κB, a central transcription factor that drives the expression of pro-inflammatory mediators, such as TNF-α and IL-6, during microglial activation [44,45]. In our model, BCP treatment demonstrated to significantly reduce Aβ-induced phosphorylation of the NF-κB subunit p65. Consistently, such effect resulted to be almost completely abolished by co-administration of CB2R antagonist SR144528.

Beyond the involvement of CB2R in the anti-inflammatory action of BCP, our results also highlighted the contribution of the nuclear receptor PPARγ. Notably, we observed that the protective effect of BCP on NF-κB signaling was reversed by co-administration of PPARγ antagonist GW9662, indicating that the activation of PPARγ may also contribute to the BCP anti-inflammatory response. Consistently, Cheng et al. (2014) have previously demonstrated that in APP/PS1 mice BCP neuroprotection was abolished by pharmacological inhibition of either CB2R or PPARγ, suggesting a functional interdependence between the two pathways [46] in the action of BCP.

Even though BCP is not considered a direct ligand of PPARγ [14], previous studies have suggested that the activation of PPARγ may occur indirectly, as a downstream consequence of CB2R stimulation by BCP [14,29]. This is supported by evidence showing that CB2R activation can initiate intracellular signaling cascades (e.g., PI3K/Akt, PGC-1α/SIRT1) that promote PPARγ activity and gene expression [31,32]. Once activated, PPARγ can inhibit NF-κB through multiple mechanisms, including direct interaction with the p65 subunit, competition for transcriptional coactivators, and upregulation of IκBα [46,47,48].

This dual involvement of CB2R and PPARγ in mediating BCP effects has also been reported in models of systemic inflammation and metabolic dysfunction, including colitis, dyslipidemia, and collagen-induced arthritis [20,49]. In these conditions, BCP has been shown to reduce inflammation and tissue damage through concurrent activation of both CB2R and PPARγ.

**Figure 9:**
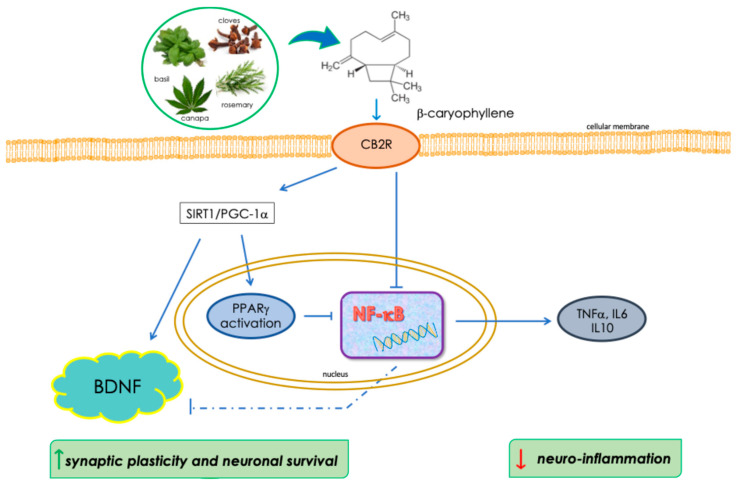
Schematic representation of BCP/CB2R/PPARγ system.

Based on this cumulative evidence, we propose that a dual mechanism involving both CB2R and PPARγ and converging on the inhibition of NF-κB signaling may contribute to the anti-inflammatory effects of BCP in microglia (Figure 9). Moreover, BCP effectively prevented Aβ-induced reductions in BDNF levels, supporting a broader mechanism of action that involves neuronal survival and synaptic integrity (Figure 9). Notably, this effect was consistently observed in both human microglial cells (HMC3) and murine organotypic brain slice cultures (BSCs). BDNF is a key neurotrophin involved in neuronal repair, plasticity, and cognitive function, and its dysregulation has been widely implicated in the pathogenesis of NDDs. BDNF expression is known to be negatively affected by neuroinflammatory conditions, in which sustained NF-κB activation plays a central role. This pathway suppresses BDNF transcription both indirectly—by promoting the release of pro-inflammatory cytokines followed by impaired neurotrophin production—and directly, by competing with CREB for transcriptional coactivators like CBP/p300 [50]. Accordingly, the observed inhibition of NF-κB by BCP may also contribute to prevent the decreased expression of BDNF caused by exposure to β-amyloid.

Previous studies have shown that compounds acting on both CB2R and PPARγ, such as cannabidiol (CBD) [51,52], exert neuroprotective effects partly by enhancing BDNF expression. Accordingly, our data indicate that BCP promotes BDNF expression, potentially through the activation of key cellular pathways, including PGC-1α and SIRT1 [14], which are well-known regulators of oxidative stress, mitochondrial function, and neurotrophic signaling. As discussed above, these two factors also serve as transcriptional coactivators of PPARγ [53,54], suggesting that the observed increase in BDNF may result from an integrated signaling cascade involving CB2R stimulation, activation of PGC-1α/SIRT1 activation, and downstream engagement of PPARγ.

Moreover, BDNF upregulation by BCP has been recently reported in preclinical models of cognitive impairment [55,56]. Therefore, BDNF restoration by BCP may represent a key downstream event contributing to its neuroprotective and pro-cognitive effects in Aβ-induced neurotoxicity models. By performing electrophysiological experiments focusing on the entorhinal cortex (EC), a crucial site for the development of amyloid-dependent neurodegeneration [14,29], we provided functional evidence of the impact of BCP on synaptic plasticity, a key determinant of cognitive dysfunction in AD [30]. Indeed, we showed that Aβ_1-42_ inhibition of LTP in EC was prevented by the application of BCP at 10 μM concentration, a dosage that we have previously shown to be able to restore Aβ_1-42_ dependent decreased BDNF levels in mouse BSCs. Notably, it is widely accepted that high levels of BDNF are associated with lower risk of cognitive impairment in AD patients, and most AD drugs currently used in clinical or still under development increase BDNF biosynthesis [57,58,59].

In conclusion, the results of our study suggest a pleiotropic mechanism of action for the development of BCP neuroprotective effects in relation to amyloid-induced neuroinflammation and synaptic impairment, encouraging further investigations into an in vivo model of amyloid-dependent cognitive damage to clarify the exact mechanism of action of BCP and confirm whether this natural molecule may represent a novel option for the treatment of NDDs.

Furthermore, the potent anti-inflammatory effects exerted by BCP through the interaction of CB2 and PPARγ receptors support the therapeutic potential of BCP in a broad range of conditions, including neurodegenerative and metabolic diseases, neuropathic pain, and cancer [60,61]. Taking into consideration the safety of BCP in humans, dietary use, and its efficacy in various experimental models of disease, BCP may be further explored as co-supplementary drug in experimental studies.

## 4. Materials and Methods

### 4.1. Test Compounds

BCP was commercially purchased (22075, Sigma Aldrich, Milan, Italy) and stored at 4 °C.

CB2R antagonist SR144528 and PPAR-γ antagonist GW9662 were commercially purchased (SML1899, M6191, respectively, Sigma Aldrich, Milan, Italy) and dissolved in DMSO (D2650, Sigma Aldrich, Milan, Italy). Aliquots were stored at −20 °C.

Before the experiments BCP stock solution was diluted into the cell culture medium to the desired experimental concentration, and the final DMSO concentration was maintained no higher than 0.1%. Vehicle-treated cells (0.1% DMSO) were used as control.

In all the experiments 24 h after seeding, cells were exposed to pretreatment with BCP (10 and 25 µM) for 24 h and then exposed to Aβ_25-35_ at the pertinent concentration (1 µM for HMC3, 10 µM for U87-MG). After 48 h, cells were processed according to the specific experiment protocol. Vehicle-treated cells were used as control. In competition experiments, the CB2R antagonist SR144528 (1 µM) and the PPAR-γ antagonist GW9662 (20 µM) were administered 15 min before proceeding with the administration of the test compounds.

### 4.2. Cell Cultures and Reagents

Human microglial clone 3 (HMC3, ATCC^®^ CRL-3304TM, Manassas, VA, USA) cell lines were cultured in EMEM supplemented with 10% fetal bovine serum and a 1:1 antibiotic mixture of streptomycin (100 g/mL) and penicillin (100 U/mL) (Sigma-Aldrich, Milan, Italy) at 37 °C in 5% CO_2_ humidified air.

Aβ_25-35_ (A4559, Sigma-Aldrich, Milan, Italy) was initially dissolved in double-distilled water to obtain 1 mM concentration and stored at −20 °C. To form aggregated diffusible oligomers, the solution was incubated at 37 °C for 5 days [62], then diluted in medium to the indicated concentration, just prior to cell treatments.

Aβ_1-42_ peptide was purchased from Biosource (Camarillo, CA, USA). Aliquots were stored at −20 °C in DMSO as a 200 μM stock solution and diluted to the desired final concentration in culture medium, immediately before application [36].

### 4.3. MTT Assay (Cell Viability Assay)

Cells were exposed to cell viability assays by using 3-(4,5-dimethylthiazol-2-yl)-2,5- diphenyltetrazolium bromide (MTT) reagent (M5655, Sigma Aldrich, Milan, Italy). Briefly, cells were exposed to increasing concentrations of BCP (5–400 μM) and incubated at 37 °C for 24 h. Then, 0.5 mg/mL MTT reagent was added to each well, and the cells were incubated for 4 h at 37 °C. The formazan products were dissolved in DMSO. After incubating for 10 min at 37 °C, absorbance at 540 nm was determined with an automated microplate reader (BIO-TEK, Winooski, VT, USA). The percentage of cell viability was calculated as a percentage of vehicle-treated cells used as control. The same procedure was also followed to detect the cytotoxic effect produced in cells after incubation with Aβ_25-35_ in the absence and presence of BCP (5, 10, 25 μM).

### 4.4. Gene Expression Analysis

Total RNA was extracted using the RNeasy Mini kit (74104, Qiagen, Hilden, Germany) following manufacturer protocol. Qubit v.1 fluorometer plus Qubit RNA HS Assay Kit (Thermo Fisher Scientific, Wilmington, DE, USA) was used to quantify total RNA.

Total RNA (1 µg) was retrotranscribed into first-strand cDNA by using iScriptTM gDNA Clear cDNA Synthesis Kit (Bio-Rad, Milan, Italy) according to the manual protocol indications. The obtained cDNA samples were measured by real-time PCR using a SYBR Green probe and an CFX Connect Real-Time PCR Detection System (Bio-Rad, Milan, Italy). The PCR cycle program consisted of an initial denaturation at 95 °C for 30 s, followed by 40 cycles of 5 s denaturation at 95 °C and 15 sec annealing/extension at 60 °C. A final melting protocol with ramping from 65 °C to 95 °C with 0.5 °C increments of 5 s was performed to verify the amplicon specificity and primer dimer formation.

Primer sequences were reported in Table 1. All reactions were performed in duplicate and the amount of mRNA was calculated by the comparative CT method. To account for possible variations related to cDNA input or the presence of PCR inhibitors, the endogenous reference gene GAPDH was quantified for each sample, and data normalized accordingly.

### 4.5. Animals

C57BL/6 wild-type mice were obtained from Jackson Laboratories (Bar Harbor, ME, USA), and were bred in the CNR Institute of Neuroscience’s animal facility. The experiments were performed in male animals of 4–5 months. Mice were kept under a 12 h dark to light cycle (from 7.00 a.m. to 7.00 p.m.), with food and water ad libitum. All experiments with mice were performed according to the national and international laws for laboratory animal welfare and experimentation (EU directive n. 2010/63/EU and Italian DL n. 26 4 March 2014) and Ministry of Health (129/2000-A, 13 December 2000).

### 4.6. Brain Horizontal EC Slices Preparation

Animals were deeply anesthetized using urethane (20% solution, 0.1 mL/100 g of body weight) via intraperitoneal injection and then decapitated after disappearance of the tail pinch reflex. The brain was rapidly removed, and thick horizontal sections (400 µm for electrophysiological recordings; 300 µm for organotypic cultures) containing the EC were made on a Vibratome Leica VT1200S (Leica Biosystems, Milan, Italy). All steps were performed in ice-cold artificial cerebrospinal fluid (ACSF) containing mM: NaCl, 119; KCl, 2.5; CaCl_2_, 2; MgSO_4_, 1.2; NaH_2_PO_4_, 1; NaHCO_3_, 6.2; D-glucose 11; HEPES, 10; pH 7.4. All reagents used for ACS preparation (inorganic salts, glucose, and HEPES) were purchased from Sigma-Aldrich (Merck, Darmstadt, Germany), ≥99% purity, ACS grade. The solution was oxygenated by an oxygenator (AirSep, Buffalo, NY, USA).

### 4.7. Organotypic Cultures

EC brain slices were transferred onto 30 mm diameter semi-porous membrane inserts (Millicell-CM PICM03050; Millipore, Vimodrone, Italy), which were placed in six-well tissue culture plates containing 1.2 mL of culture medium. The culture medium consisted of 50% Eagle’s minimal essential medium, 25% heat-inactivated horse serum, 25% Hanks’ balanced salt solution, 5 mg·mL^−1^ glucose, 2 mM L-glutamine, and 3.75 mg/mL amphotericin B. Slices were incubated at 37 °C in an atmosphere of humidified air and 5% CO_2_ for 24 h. Then, *n* = 3 slices were exposed to 1 μM β-amyloid peptide 1-42 (Aβ_1-42_) for 24 h; *n* = 4 slices were pretreated with 10 μM BCP and then exposed to 1 μM Aβ_1-42_ for 24 h; *n* = 4 slices were pretreated with 25 μM BCP for 4 h and then exposed to 1 μM Aβ_1-42_ for 24 h; in the same set of experiment, *n* = 3 slices were maintained in regular growth medium, and used as control. The experiment was conducted according to the protocol described in Gerace et al., 2012 [63]. For all the groups, at the end of the incubation period, media were collected to assay the release of common pro-inflammatory cytokines and BDNF by specific ELISA assays.

### 4.8. In Vitro Electrophysiology

Field excitatory postsynaptic potentials (fEPSPs) were evoked by a concentric bipolar stimulating electrode in layer II/III of EC, as previously reported [36,64]. The recording electrode was obtained from borosilicate capillaries (OD. 1.0 mm, ID 0.78 mm) using a puller (PP 830, Narishige) and was placed in layer II/III of EC. The amplitude of the fEPSPs was used as a measure of the evoked population excitatory current. The change in the amplitude of fEPSPs reflects the change in the slope in the negative deflection of the recorded post-synaptic potential and correlates with changes in the source of monosynaptic currents. The amplitude of the negative deflection of the extracellular potential was used as a measure of the population of the excitatory currents that are evoked by the stimulus. The stimulus was applied by an isolator (Digitimer DS2A; Digitimer Ltd., Welwyn Garden City, Hertfordshire, UK) and baseline responses were obtained with a stimulation intensity that yielded 50–60% of maximal amplitude. All fEPSPs had peak latency between 4.5 and 6 ms. FEPSPs’ amplitude was monitored every 20 s and averaged every three responses by an online data acquisition software. After 15 min of stable baseline, recording high frequency stimulation (HFS, three trains of 100 pulses at 100 Hz, 10 s interval) was used to induce long term potentiation (LTP). LTP magnitude was measured as the average of fEPSPs amplitudes between the 40th and the 50th min after HFS. Values were expressed as mean ± SEM percentage change relative to their mean baseline amplitude [65]. Aβ_1-42_ (200 nM) and/or BCP (10 µM) were applied through general perfusion for 10 min starting from 5 min before HFS. ACSF-perfused slices were used as control.

### 4.9. ELISA Assays

Pro-inflammatory IL-6 and anti-inflammatory IL-10 levels were evaluated by specific ELISA assays [RAB0306 (IL-6), RAB0244 (IL-10), Sigma-Aldrich, Milan, Italy] on collected culture media from HMC3 cells. In addition to IL-6 and IL-10 measurements, the levels of tumor necrosis factor TNFα (RAB1089, Sigma-Aldrich, Milan, Italy) and nuclear factor-kB (NF-kB) (85-86082-11, ThermoFisher Scientific, Carlsbad, CA, USA) were also evaluated in collected culture media and in cell lysates, respectively.

The release of TNFα [RAB0477, Sigma-Aldrich, Milan, Italy] and IL6 [RAB0308, Sigma-Aldrich, Milan, Italy] was also analyzed on collected media from mouse BSCs, following the corresponding manufacturer’s instructions.

Brain-derived neurotrophic factor (BDNF) (211BEK-2211-1P, BSENBEK, Biosensis, Thebarton, South Australia, Australia) levels were evaluated on collected culture media either from microglia cells and mouse BSCs. Before characterization, mouse BSCs were homogenized in cold lysis buffer (1% NP-40, 20 mM Tris–HCl pH 8, 130 mM NaCl, 10 mM NaF, 10 μg/mL aprotinin, 10 μg/mL leupeptin, 40 mM DTT, 1 mM Na_3_VO_4_, and 10 mM PMSF) by the use of a homogenizer prechilled on ice. Homogenates (20 µg) were kept on ice for 30 min and centrifuged at the maximum speed for 15 min; the supernatant was collected, and the concentrations of BDNF in homogenized extracts were measured with commercial ELISA kits in 96-well strip plates. All reagents and standard dilutions were prepared following the manufacturer’s instructions. Absorbances were measured in a microplate reader (Tecan Infinite 200PRO, Tecan, Männedorf, Switzerland) at 450 nm, and the results were calculated using a standard curve following the manufacturer’s instructions.

### 4.10. Statistical Analysis

All data are reported as the mean ± standard error of the mean (SEM). Statistical analyses were performed with commercial software (GraphPad Prism v.10, San Diego, CA, USA) using ordinary one-way ANOVA followed by Dunnett’s or Tukey’s post hoc tests. Differences for which *p* < 0.05 were considered significant.

## Figures and Tables

**Table 1:** Primer sequences for real-time PCR experiments.

Reference Sequence RNA	Gene Symbol	Forward Primer	Reverse Primer
NM_001354667.3	PPARg	AGCCTGCGAAAGCCTTTTGGTG	GGCTTCACATTCAGCAAACCTGG
NM_001142498.2	SIRT1	TAGACACGCTGGAACAGGTTGC	CTCCTCGTACAGCTTCACAGTC
NM_013261	PGC-1a	CCAAAGGATGCGCTCTCGTTCA	CGGTGTCTGTAGTGGCTTGACT
NM_170734	BDNF	CATCCGAGGACAAGGTGGCTTG	GCCGAACTTTCTGGTCCTCATC
NM_002046	GAPDH	GTCTCCTCTGACTTCAACAGCG	ACCACCCTGTTGCTGTAGCCAA

## Data Availability

The original contributions presented in this study are included in the article. Further inquiries can be directed to the corresponding authors.
